# Fine-grained weed recognition using Swin Transformer and two-stage transfer learning

**DOI:** 10.3389/fpls.2023.1134932

**Published:** 2023-03-13

**Authors:** Yecheng Wang, Shuangqing Zhang, Baisheng Dai, Sensen Yang, Haochen Song

**Affiliations:** ^1^ College of Engineering, Northeast Agricultural University, Harbin, China; ^2^ College of Electrical Engineering and Information, Northeast Agricultural University, Harbin, China; ^3^ College of Agriculture, Northeast Agricultural University, Harbin, China

**Keywords:** deep learning, fine-grained weed recognition, Swin Transformer network, contrastive loss, transfer learning

## Abstract

Weeding is very critical for agriculture due to its importance for reducing crop yield loss. Accurate recognition of weed species is one of the major challenges for achieving automatic and precise weeding. To improve the recognition performance of weeds and crops with similar visual characteristics, a fine-grained weed recognition method based on Swin Transformer and two-stage transfer learning is proposed in this study. First, the Swin Transformer network is introduced to learn the discriminative features that can distinguish subtle differences between visually similar weeds and crops. Second, a contrastive loss is applied to further enlarge the feature differences between different categories of weeds and crops. Finally, a two-stage transfer learning strategy is proposed to address the problem of insufficient training data and improve the accuracy of weed recognition. To evaluate the effectiveness of the proposed method, we constructed a private weed dataset (MWFI) with maize seedling and seven species of associated weeds that are collected in the farmland environment. The experimental results on this dataset show that the proposed method achieved the recognition accuracy, precision, recall, and F1 score of 99.18%, 99.33%, 99.11%, and 99.22%, respectively, which are superior to the performance of the state-of-the-art convolutional neural network (CNN)-based architectures including VGG-16, ResNet-50, DenseNet-121, SE-ResNet-50, and EfficientNetV2. Additionally, evaluation results on the public DeepWeeds dataset further demonstrate the effectiveness of the proposed method. This study can provide a reference for the design of automatic weed recognition systems.

## 1 Introduction

Weeds are undesirable plants in the field because they usually compete with early-growth crops in essential resources, such as light, water, and nutrients. If weeds are not controlled in time, they will cause severe threats to food security by reducing crop production. According to the estimation, approximately 34% of all crop losses globally are due to weeds (Oerke, 2006). Chemical weed control is the most common and effective weeding form used by farmers in the current period (Westwood et al., 2018). However, chemical weeding results in serious herbicide waste and potential ecological pollution. In addition, large doses of herbicide spraying can cause some weeds to develop resistance, making them difficult to remove weeds completely (Hawkins et al., 2019). To reduce the negative impact of herbicides, it is necessary to implement precise weeding, i.e., site-specific application of herbicides. Automatic weed recognition based on computer vision technology can provide accurate weed distribution information for weed control systems, which is the primary prerequisite for precise weeding (López-Granados, 2011).

Early computer vision methods for automatic weed recognition relied on hand-crafted features such as shape and texture. The combination of these features with machine learning algorithms (e.g., support vector machines and artificial neural network) has yielded good results in weed recognition (Tang et al., 2003; Guerrero et al., 2012; Behmann et al., 2015). However, due to the limited ability of manual features to distinguish weed species, the above methods only performed well on weed images with large morphological differences and were difficult to accurately identify weeds and crops in farmland environments. Benefiting from significant advances in deep learning, convolutional neural network (CNN) has achieved good performance in agriculture image analysis (Saleem et al., 2021). Recently, the researchers attempt to utilize CNN in weed recognition for its powerful capability of feature learning. Dyrmann et al. (2016) built a CNN-based model, i.e., ResNet, for classifying weed and crop species. dos Santos Ferreira et al. (2017) directly used the AlexNet model to identify soybean and its major associated weeds. Olsen et al. (2019) constructed the DeepWeeds dataset, which is a multi-class weed dataset containing 17,509 images and fine-tuned InceptionV3 and ResNet-50 directly on this dataset to recognize weeds in rangelands. Espejo-Garcia et al. (2020) fine-tuned pre-trained CNN (Xception, Inception-Resnet, VGGNet, Mobilenet, and Densenet) to extract weed features and combined extracted features with machine learning classifiers to identify weeds. Some recently emerged deep learning techniques, such as graph convolutional network (GCN) and generative adversarial network (GAN), are also employed to assist in weed recognition. Jiang et al. (2020) employed GCN to enrich extracted weed features and acquired high performance on four different weed datasets. Espejo-Garcia et al. (2021) created amounts of synthetic weed images through GAN for weed recognition.

**Figure 1:**
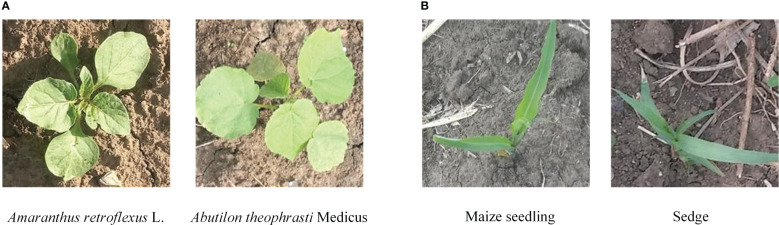
Example images belonging to different categories of crop and weed in the field. **(A)** Different categories of weeds with similar appearance. **(B)** Weed and crop with similar appearance.

Weed recognition in the field belongs to fine-grained image recognition issue due to the lack of obvious visual characteristics between a portion of sub-categories. For example, as shown in **Figure 1**, in the weed recognition task, there exist low interclass variance between weeds and crops due to the fact that their leaves share similar attributes. Thus, it is a quite challenging task to deep-learning methods achieving high-precision performance for fine-grained weed recognition in field conditions. Most previous works directly captured features in the whole image, without adequately considering similar visual characteristics between weeds and crops. Although the deep model can accurately distinguish image samples with the complex background, illumination change, deformity, and occlusion, the similar visual appearance and texture between different categories became the main cause of recognition errors (Ferreira et al., 2017; Jiang et al., 2020). Hu et al. (2020) proposed a graph weeds network (GWN) for recognizing multiple types of weeds; this method can precisely capture discriminative weed features by formulating weed images as multi-scale graph representations, which achieved 98.1% accuracy on the DeepWeeds dataset. However, the architecture of GWN has low efficiency, thus making it difficult to achieve real-time weed recognition. Rzanny et al. (2022) evaluated different combinations of CNN-based feature extraction and classifiers to distinguish 31 Poaceae species with similar morphology; this work, through combinations of six perspective images as input to provide more information about weed species, achieved the best accuracy of 96.1%. However, it is difficult to acquire multiple perspective images in weed control operations.

Vision Transformer (ViT) is an effective approach to solving fine-grained image recognition problems (He et al., 2022; Zhang et al., 2022). It applies self-attention mechanisms directly to image patch sequences to capture important regions in images, and finally learns significant features to improve the classification performance (Dosovitskiy et al., 2020; Han et al., 2022). However, the self-attention in the transformer is quadratic to the image size, and directly applying pure transformer to weed recognition has a high requirement for computational resources. The Swin Transformer is one of the variants of ViT, which computes self-attention within small windows to model local relationships and uses a window shift strategy to gradually enlarge the receptive field, and can learn more powerful and robust representations than CNN (Liu et al., 2021). Therefore, it is suitable for fine-grained weed recognition where there are subtle morphological differences between weeds and crops.

Considering the characteristics of the visual similarity between weeds and crops, the Swin Transformer network is introduced to learn fine-grained feature representation. Meanwhile, to further enlarge the feature differences between different categories of weeds and crops, a combination of contrastive loss and cross-entropy loss is applied to guide model optimization. In addition, by adopting a two-stage transfer learning strategy, the deep network can alleviate the dependencies of a large amount of labeled data and is expected to achieve higher accuracy. The main contributions of our work can be summarized as follows:

(1) A fine-grained recognition method based on Swin Transformer is proposed for weed recognition and a contrastive loss is applied to improve the ability to distinguish between weeds and crops. This method achieves state-of-the-art performance on our private MWFI dataset and public DeepWeeds dataset.
(2) A two-stage transfer learning strategy is proposed to solve the problem of insufficient training data and improve weed recognition accuracy.
(3) Evaluation results on MWFI and DeepWeeds datasets demonstrate the ability of the proposed method to accurately recognize weeds and crops with similar morphology.
(4) To the best of our knowledge, this study is the first to introduce the Swin Transformer network to learn fine-grained features of weeds and crops, which offers an alternative to the dominating CNN-based architectures.

The rest of this study is organized as follows. *Section 2* introduces our used datasets. *Section 3* elaborates the proposed fine-grained weed recognition method. *Section 4* describes the experimental setting. *Section 5* reported experimental results and discussion. Finally, we conclude the study and give our future work in *Section 6*.

## 2 Datasets

### 2.1 MWFI dataset

**Figure 2:**
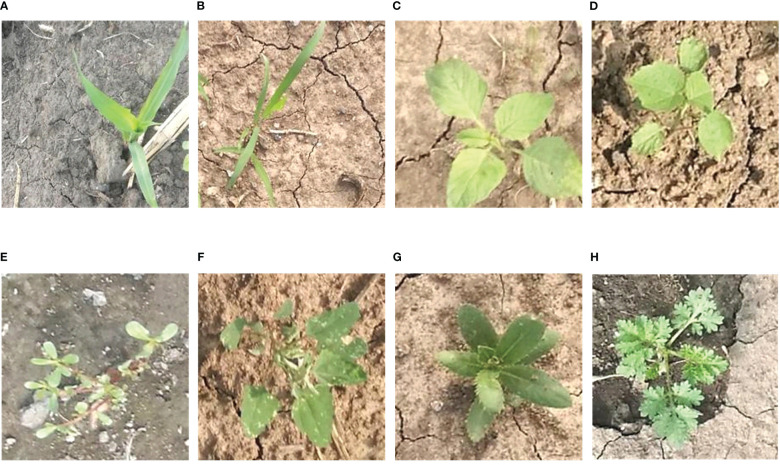
Example images of maize seedling and weeds in the MWFI dataset. **(A)** Maize seedling, **(B)** *Cyperus rotundus* L., **(C)** *Amaranthus retroflexus* L., **(D)** *Abutilon theophrasti* Medicus, **(E)** *Portulaca oleracea* L., **(F)** *Chenopodium album* L., **(G)** *Cirsium setosum*, and **(H)** *Descurainia sophia* (L.)Webb. ex Prantl.

The images in the MWFI (maize/weed field image) dataset were taken from the maize field at Northeast Agricultural University located in Harbin, Heilongjiang Province, China (126° 43′ 39″ E, 45° 44′ 38″ N). All images were vertically captured 60 cm above the ground by a smartphone camera. Considering the importance of weed recognition at the early growth stage, the image acquisition was conducted from 25 May 2021 to 5 June 2021 when maize is in the growth stage of two to five leaves. When capturing images, only one kind of weed appeared in the approximate middle of the images to ensure that each image contains one target weed species. The captured images had a resolution of 640 × 480 pixels. To reduce the computational burden of the deep learning-based methods, the original images are center cropped to 400 × 400 pixels to ensure the same aspect ratio and then resized it to 224 × 224 pixels by bilinear interpolation algorithm to build the dataset. The constructed dataset contains 1,632 images belonging to a crop (Maize seedling) and its associated weeds [*Cyperus rotundus* L., *Amaranthus retroflexus* L., *Abutilon theophrasti* Medicus, *Portulaca oleracea* L., *Chenopodium album* L., *Cirsium setosum* and *Descurainia sophia* (L.)Webb. ex Prantl]. Example images of maize seedling and weeds are shown in **Figure 2**. This dataset is divided into 1,142 training images and 490 testing images by a ratio of 7:3.

In this work, data augmentation techniques are adopted to improve the generalization ability of the method and address the impact of uneven sample distribution on recognition performance. Specifically, the number of samples of each category in the training set was expanded by using brightness enhancement, random rotation, adding salt and pepper noise, and Mosaic (Bochkovskiy et al., 2020; Hasan et al., 2021). Example results of data augmentation are shown in **Supplementary Figure 1**. **Supplementary Table 1** summarizes the full names of each category, the abbreviation of each category, and the number of training and testing images.

### 2.2 Plant seedlings dataset

The Plant Seedlings dataset includes 5,539 images of 12 plant species (Giselsson et al., 2017), which were acquired from plant seedlings belonging to three crops (maize, common wheat, sugar beet) and nine weeds (*Alopecurus myosuroides*, *Sinapis arvensis* L., *Galium aparine* L., *Stellaria media, Chenopodium album* L., *Polygonum persicaria* L., *Matricaria perforata M´erat*, *Capsella bursa-pastoris*, and *Geranium pusillum*). Because all images of this dataset with simple backgrounds were taken under controlled laboratory conditions, it is easy to achieve the high accuracy of weed recognition on this dataset. Therefore, the Plant Seedlings dataset is selected as an additional dataset for two-stage transfer learning.

## 3 The proposed method

**Figure 3:**
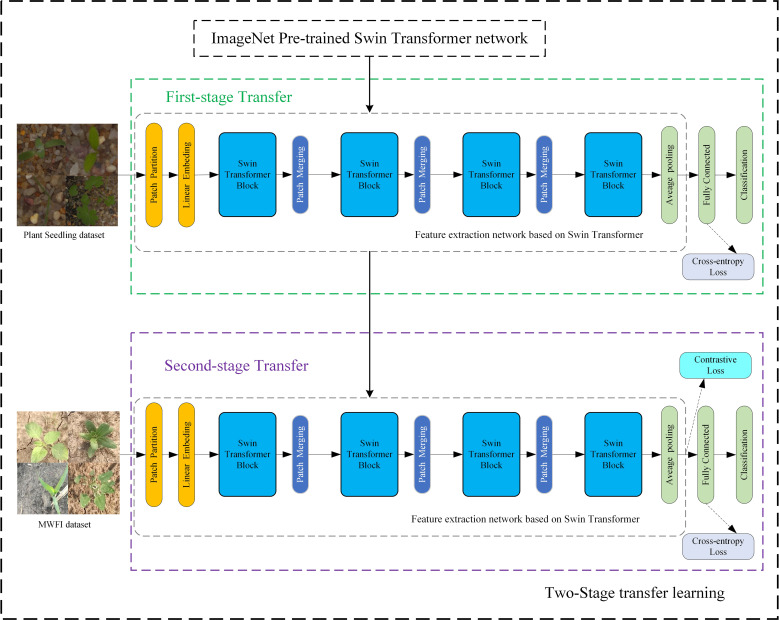
The proposed fine-grained weed recognition method.

This section describes the details of the proposed method of fine-grained weed recognition. To accurately distinguish maize seedlings and weeds with high appearance similarity, a Swin Transformer network was introduced for the feature extraction network and a contrastive loss was applied to expand the feature differences between maize seedlings and weeds in this method. The overall structure is illustrated in **Figure 3**; this method mainly consists of two stages. In the first stage, a Swin Transformer network is fine-tuned on the Plant Seedlings dataset to obtain a task-related pre-trained network. The second stage aims to fine-tune the task-related pre-trained network on our MWFI dataset and apply a contrastive loss to assist network training.

### 3.1 Feature extraction network

**Figure 4:**
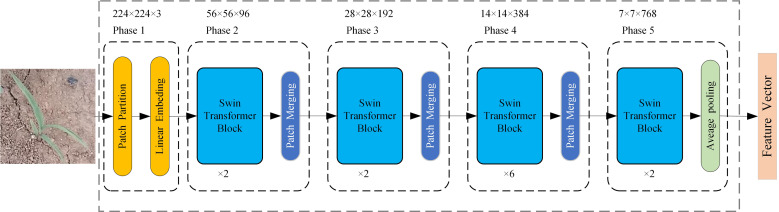
The feature extraction network based on Swin Transformer.

The feature extraction network based on Swin Transformer consists of five phases (Liu et al., 2021), as shown in **Figure 4**. The network input is an RGB three-channel weed image of 224 × 224 × 3 size. In phase 1, the input image is divided into 4 × 4 × 3 non-overlapping image patches by a patch partition module, and then the set of image patches is projected into multi-channel single-pixel points by a linear embedding layer. These two operations are realized by convolution with a kernel size of 4 × 4, an output channel of 96, and a step size of 4. The input image becomes a 56 × 56 × 96 feature map after phase 1.

**Figure 5:**
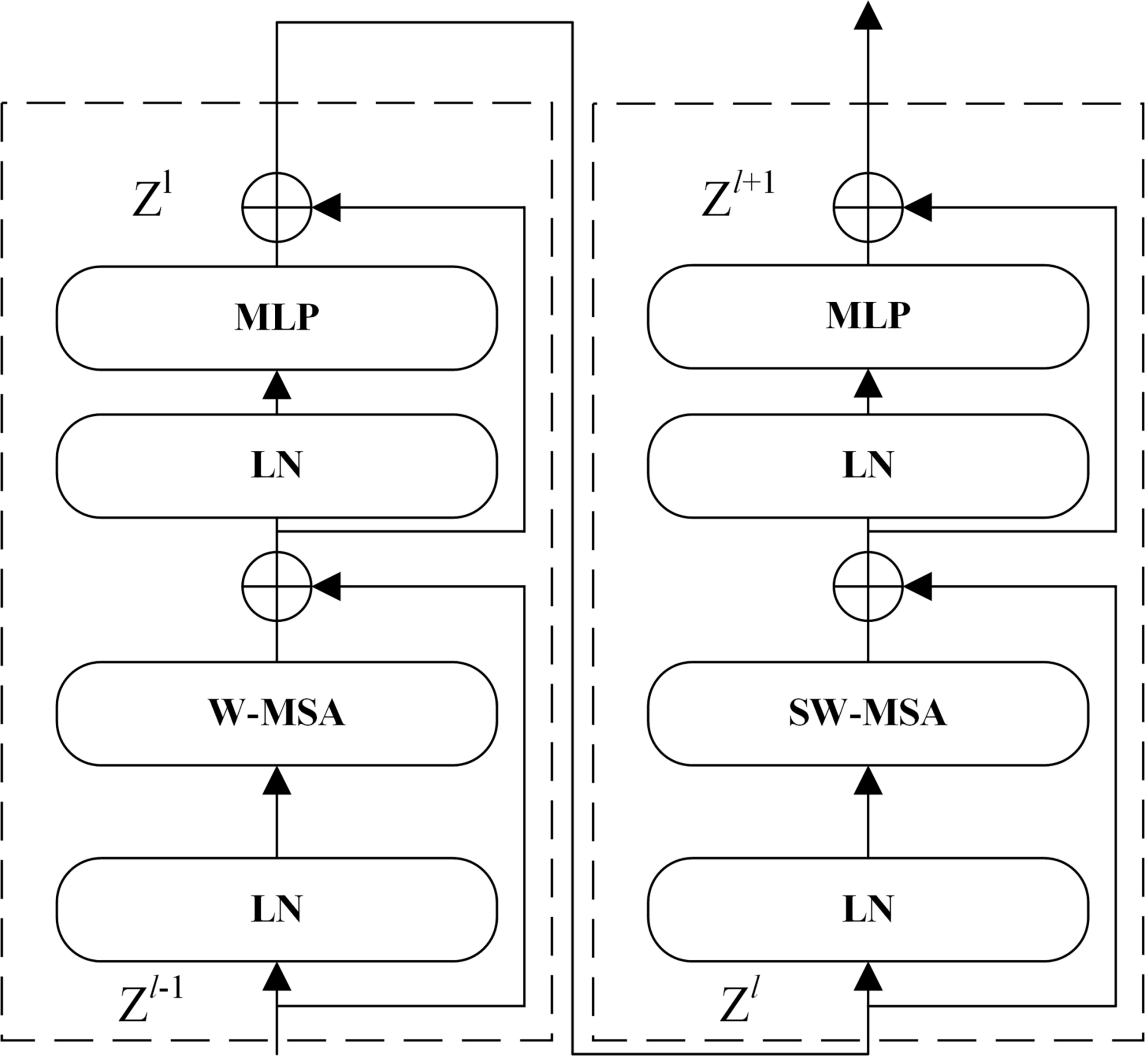
Two successive Swin Transformer blocks.


In phase 2, the feature map is sent to multiple stacked Swin Transformer blocks for feature extraction and then downsampled by a patch merging layer to generate a hierarchical feature representation. Two consecutive Swin Transformer blocks are applied for feature transformation to ensure that the network learns global semantic features and local semantic features at the same time. As shown in **Figure 5**, two consecutive Swin Transformer blocks are made up of window-based multi-head self-attention (W-MSA), shifted window-based multi-head self-attention (SW-MSA), and multilayer perceptron (MLP) that includes two fully connected layers and a GELU nonlinearity. Specifically, a residual connection is used after each (S)W-MSA module and each MLP, and a LayerNorm (LN) layer is used before each module. The computations of two consecutive Swin Transformer blocks can be formulated as follows:

(1) $${\text{Z}}^{l}\;=\text{ MLP(LN(W}-{\text{MSA(LN(Z}}^{l-1}\text{)) }+{\text{ Z}}^{l-1}{\text{)) + W-MSA(LN(Z}}^{l-1}{\text{)) + Z}}^{l-1}$$

(2) $${\text{Z}}^{l+1}\;={\text{ MLP(LN(SW-MSA(LN(Z}}^{l}\text{)) }+{\text{ Z}}^{l}\text{)) }+\text{ SW}-{\text{MSA(LN(Z}}^{l}{\text{)) + Z}}^{l}$$

where Z*^l^* ^-1^ is the input features of the W-MSA module, Z*^l^* represents the output features of the W-MSA module and the MLP module for block *l*, and Z*^l^* ^+1^ represents the output features of the SW-MSA module and the MLP module for block *l*+1.

**Figure 6:**
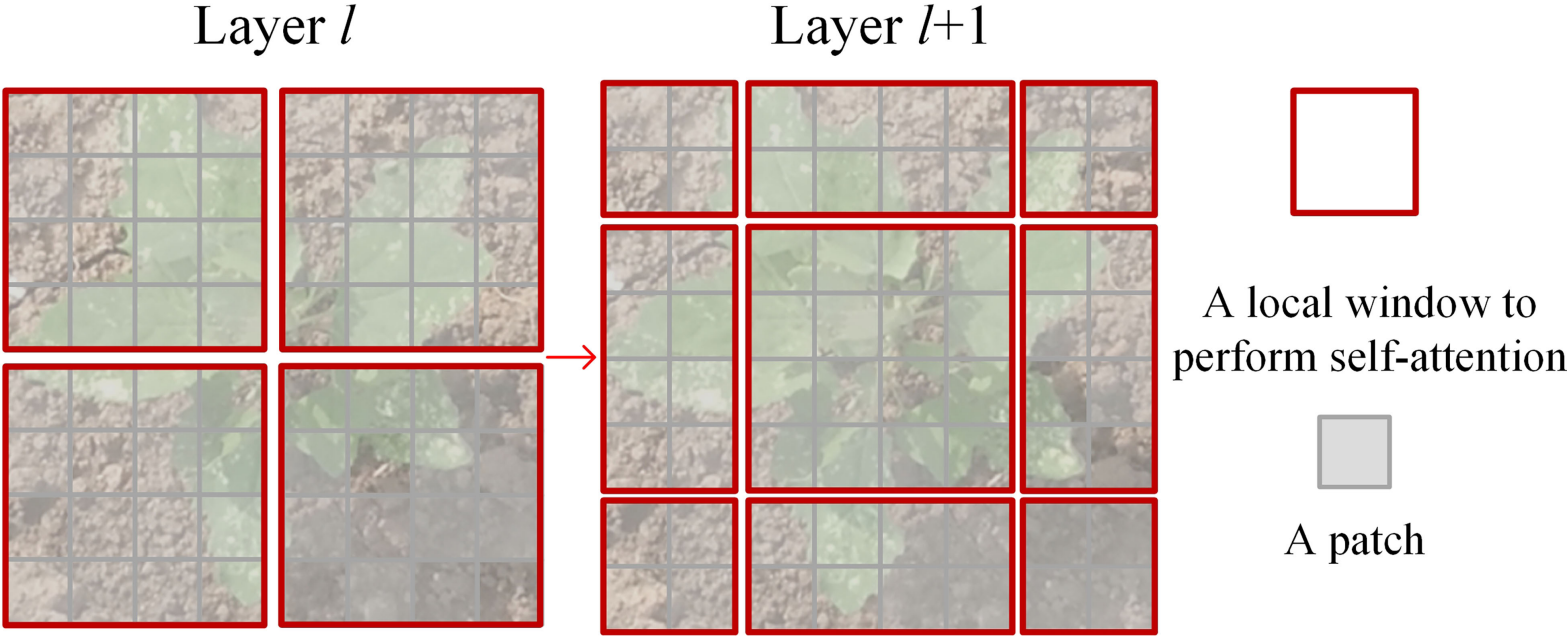
An illustration of the shift window approach for computing self-attention in the Swin Transformer architecture.

The W-MSA module is used to compute self-attention within local windows in which each window contains *M* × *M* patches (*M* = 7). The W-MSA module significantly reduces the computation compared with the standard multi-head self-attention (MSA). The comparison of computational complexity between the MSA and W-MSA is as follows:


(3) $$\Omega \text{(MSA)}=4hw{\text{C}}^{2}+\text{2(}hw{)}^{2}\text{C}$$

(4) $$\Omega \text{(W}-\text{MSA)}=4hw{\text{C}}^{2}{+2M}^{2}hw\text{C}$$

where *h* represents the height of the input feature map, *w* represents the width of the input feature map, and *C* represents the depth of the input feature map. The W-MSA module that has linear computational complexity relative to image size is affordable for the weed recognition tasks. Since W-MSA is only a local attention mechanism, the SW-MSA module is proposed to introduce cross-window communication, as shown in **Figure 6**. The SW-MSA module through the shift window approach offers the connections of feature maps that belong to neighboring non-overlapping windows to capture global information. It is effective for image classification.

Since the self-attention mechanism cannot learn the positional relationship between patches, i.e., disturbing the input order of image patches will not affect the recognition results, positional encoding is necessary to add positional information for patches. Following Liu et al. (2021) positional encoding method, relative position bias is introduced when calculating the similarity between the query and the key. Self-attention in Swin Transformer Block is computed as follows:


(5) $$\text{Attention(}Q,K,V)=\text{SoftMax(}QK^{T}/\sqrt{d}+B)V$$

where *Q, K*, and *V* represent the query, key, and value metrics; *d* is the dimension of the query/key (*d* = 32); and *B* is the relative position bias, which is used to characterize the position information between image. The patch merging layer is similar to a pooling operation. The 56 × 56 × 96 feature maps are merged in 2 × 2 adjacent blocks, resulting in a feature map with a size of 28 × 28 and a dimension of 384, and then the feature dimension is reduced to 192 by a fully connected neural network layer, thus halving the feature map size and doubling the number of channels for downsampling. Phases 3 and 4 are the same as phase 2, and the feature map size becomes 7 × 7 × 768 after phases 3 and 4. Phase 5 consists of two consecutive Swin Transformer blocks and a global average pooling layer, and outputs a comprehensive feature representation with a dimension of 768.

### 3.2 Loss function

Since the morphology differences in some weeds and maize seedlings are small, a commonly used cross-entropy loss (*L_cross_*) is not sufficient to fully supervise the learning of discriminative features. Therefore, a contrastive loss (*L_con_*) was introduced to assist the *L_cross_* to extract more discriminative features (He et al., 2022). The contrastive loss is calculated based on cosine similarity between each batch of output features that can expand the difference corresponding to the features of different categories and increase the similarity with features of the same category. Meanwhile, to reduce the contrastive loss influenced by different categories of samples with little similarity, a constant degree of similarity *α* is introduced in which only two different categories of samples with similarity greater than *α* act on the *L_con_*. The contrastive loss is defined as:


(6) $$L_{con}=\frac{1}{N^{2}}\sum_{i}^{N}[\sum_{j:y_{i}=y_{j}}^{N}\left(1-Sim(z_{i},z_{j}\right)+\sum_{j:y_{i}\neq y_{j}}^{N}\left(\max(Sim(z_{i},z_{j}\right)-\alpha ),0)]$$

where *N* is the number of samples for a training batch, *z_i_* is the feature of the *i*th image sample in the batch, *z*_j_ is the feature of the *j*th image sample in the batch, *Sim*(*z_i_*, *z*_j_) is thus the dot product of *z_i_* and *z*_j_, *y_i_* is the label of the *i*th image sample and *y_j_* is the label of the *j*th image sample, and *z_i_* and *z*_j_ are pre-processed with *l*2 normalization. We enlarge the loss for different category samples with large visual similarity. This loss forces the network to focus on subtle differences between different categories as much as possible.

Finally, our network is trained using the sum *L* of *L_cross_* and *L_con_*, as shown in the following:


(7) $$L=L_{cross}(y,p)+L_{con}(z)$$

where *L* is the total loss of network training, *p* is the predicted label, and *y* is the true label.

### 3.3 Two-stage transfer learning

Transfer learning can improve the accuracy of weed recognition and reduce the computational resources and data size required to train deep neural networks (Suh et al., 2018; Espejo-Garcia et al., 2020; Xu et al., 2021). It is especially important for the Swin Transformer network that requires large-scale training data. To achieve higher recognition accuracy on our weed recognition tasks and further reduce the requirement for training data size, a two-stage transfer learning strategy was proposed for network training in this study, as shown in **Figure 3**. The input of two-stage transfer learning is a Swin Transformer network pre-trained on the ImageNet dataset. Firstly, all parameters of the Swin Transformer network before the final fully connected layer are transferred to the feature extraction network. Next, the feature extraction network is fine-tuned on the Plant Seedlings dataset to obtain a task-related pre-trained network that includes more useful knowledge for weed identification tasks. Finally, the task-related pre-trained network is again fine-tuned on our MWFI dataset to obtain the final weed recognition network.

## 4 Experiment

### 4.1 Implementation details

In this study, all the experiments are performed with NVIDIA Tesla T4 GPU and Intel(R) Core(TM) i7-12700H @ 2.70 GHz CPU using the PyTorch toolbox. The training hyper-parameter settings of seven methods in *Section 5.1* are as follows. All the networks’ input size is 224 × 224 × 3. Each deep learning network is trained for a total of 60 epochs. The batch size is set to 64. The learning rate is initialized as 0.0001. The similarity *α* in the contrastive loss is set to 0.2. The optimizer for training the Swin Transformer network and our method is AdamW, and the learning rate decayed with cosine annealing. To train CNN for comparison in *Section 5.1*, the stochastic gradient descent (SGD) optimizer with a momentum of 0.9 is adopted for network training, and the learning rate is divided by 10 after every 20 epochs.

### 4.2 Evaluation metrics

In this study, the performance of the weed recognition method is evaluated in terms of four widely used metrics: Accuracy, Precision, Recall, and F1 score, which are defined as follows:


(8) $$\text{Accuracy}=\frac{\sum_{i=1}^{c}TP_{i}}{m}\times 100\%$$

(9) $$\text{Precision}=\frac{1}{c}\sum_{i=1}^{c}\frac{TP_{i}}{TP_{i}+FP_{i}}\times 100\%$$

(10) $$\text{Recall}=\frac{1}{c}\sum_{i=1}^{c}\frac{TP_{i}}{TP_{i}+FN_{i}}\times 100\%$$

(11) $$F1 score=2\times \frac{Precision\times Recall}{Precision+Recall}$$

where *m* is the total number of samples, *c* is the number of categories, *TP* is the number of samples that are actually positive cases and classified as positive by the method, *FP* is the number of samples that are actually negative cases but classified as positive by the method, and *FN* is the number of samples that are actually positive cases and incorrectly classified as negative by the method.

To measure the real-time performance of the weed recognition method, this study uses inference time as an evaluation metric, which indicates the time required for the network to classify a single testing image by using a CPU. To measure the computational cost of the weed recognition method, the training time is also used as an evaluation metric, which indicates the total training time of two stages.

## 5 Results and discussion

### 5.1 Comparison of weed recognition performance of different methods

To verify the performance advantages of the proposed method, we compare our method against the well-performed CNN-based architectures in previous weed recognition studies including VGG-16 (Simonyan and Zisserman, 2014), ResNet-50 (He et al., 2016), DenseNet-121 (Huang et al., 2017), SE-ResNet-50 (Hu et al., 2018), EfficientNetV2 (Tan and Le, 2021), and the original Swin Transformer network. All networks are trained by using the two-stage transfer learning strategy. Network parameters corresponding to the highest accuracy on the testing set are selected for testing the performance. **Table 1** shows the experimental results.

**Table 1:** Comparison of the experimental results of different methods on the testing set.

Methods	Accuracy/%	Precision/%	Recall/%	F1 Score/%	Model sizes/MB	Inference time/ms	Training time/min
VGG-16	96.12	96.44	96.01	96.22	512	87	860
ResNet-50	96.33	96.63	96.19	96.41	90	45	270
DenseNet-121	96.73	97.02	96.78	96.90	26.9	47	990
SE-ResNet-50	96.94	97.19	97.01	97.10	147.5	61	310
EfficientNetV2	97.35	97.70	97.63	97.66	77.9	66	280
Swin Transformer	97.96	98.20	98.23	98.21	105.3	56	350
Our method	99.18	99.33	99.11	99.22	105.3	56	380

As can be seen from **Table 2**, among these comparative methods, our method achieved the best performance in terms of accuracy, precision, recall, and F1 score. Specifically, our method has a recognition accuracy of 99.19%, which is 1.83% better than EfficientNetV2 and 1.22% better than the original Swin Transformer network. The experimental results show the effectiveness of our method in weed recognition. It is also observed that the SE-ResNet-50 and EfficientNetV2 perform better than other CNN-based architectures since they can capture the discriminative feature with channel attention. Meanwhile, it can be noticed that both our method and the original Swin Transformer network exceed CNN counterparts on all evaluation metrics. This phenomenon demonstrates that the Swin Transformer network is more appropriate than CNN-based architectures for weed recognition by considering the fine-grained characteristics of weed images. In addition, the real-time performance and computational burden comparison between seven methods is shown in **Table 1**. It is observed that the inference time of our method is only 56 ms, which is similar to SE-ResNet-50 and EfficientNetV2, even better than VGG16. Our method is also less time-consuming than VGG-16 and DenseNet-121. This result shows that the Swin Transformer network does not bring a huge increase in computational complexity and our method can meet the real-time requirements of the weed recognition algorithm in weeding operations.

**Table 2:** Comparison of the recognition accuracy of different transfer learning strategies.

Methods	Without transfer learning/%	Pre-training on the ImageNet/%	Two-stage transfer learning/%
VGG-16	87.55	95.31	96.12
ResNet-50	88.97	95.71	96.33
DenseNet-121	85.71	96.12	96.73
SE-ResNet-50	86.94	95.51	96.94
EfficientNetV2	87.76	96.33	97.35
Swin Transformer	79.59	97.55	97.96
Our method	80.00	98.57	99.18

**Figure 7:**
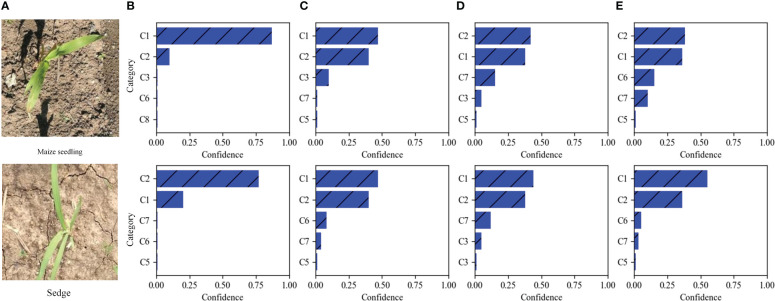
Two MWFI test images and the five categories are considered most probable by four methods. From left to right, **(A)** the test images, the results of our fine-grained weed recognition method **(B)**, EfficientNetV2 **(C)**, SE-ResNet-50 **(D)**, and DenseNet-121 **(E)**. Confidence is the prediction probability of weed recognition method for categories. C1–C8 is the abbreviation of maize seedling and weeds.

The top 5 predictions of different weed recognition methods on two test images are illustrated in **Figure 7**. It can be seen that there is a morphological similarity between the two test images. The results of recognizing the Maize seedling image are shown in the first row of **Figure 7**; DenseNet-121 and SE-ResNet-50 networks incorrectly identified Maize seedling as Sedge (i.e., *Cyperus rotundus* L.). The EfficientNetV2 network gives small differences in confidence, but correctly identified the Maize seedling. Our method not only can correctly classify the image as a Maize seedling, but also offers significant differences in confidence. The results of recognizing Sedge image are shown in the second row of **Figure 7**; DenseNet-121, SE-ResNet-50, and EfficientNetV2 networks give higher confidence to the Maize seedling than to Sedge, thus leading to the misclassification of the Sedge image. The above results show that our method can effectively learn discriminative fine-grained features through the window-based self-attention mechanism of the Swin Transformer network, and has better recognition performance in distinguishing different categories with high appearance similarity.

### 5.2 Comparison on different transfer learning strategies

To evaluate the effect of the two-stage transfer learning strategy, we compare the weed recognition performance with the different training strategies on the testing set. The result of training on the Plant Seedlings dataset can be found (**Supplementary Table 2**). As can be seen from **Table 2**, all methods have a large margin of accuracy improvement with the two-stage transfer learning strategy. It is worth mentioning that our method can improve the accuracy by 19.18% compared to that without transfer learning. The results show that two-stage transfer learning is effective for our weed recognition task. In addition, the two-stage transfer learning strategy produced higher accuracy than the network only pre-trained on the ImageNet dataset. This is because network pre-trained on Plant Seedlings dataset can provide more meaningful patterns related to maize seedlings and weeds. Therefore, it is necessary to adopt a two-stage transfer learning strategy to achieve the best recognition performance when the dataset is not large enough.

### 5.3 Evaluation of contrastive loss

We further evaluated the effect of contrastive loss on weed recognition performance. We made comparisons of the recognition accuracy with and without a contrastive loss for both EfficientNetV2 and our method on the testing set.

**Figure 8:**
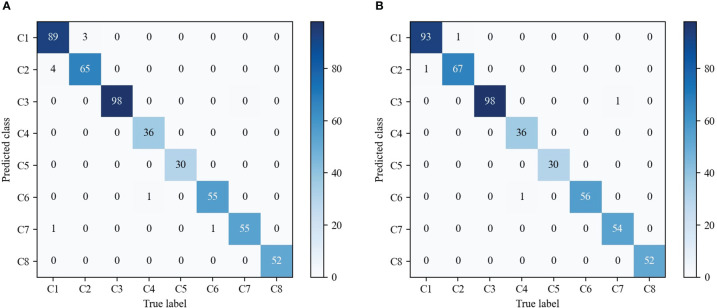
Comparison of the confusion matrices on the testing set. **(A)** Our fine-grained weed recognition method without contrastive loss. **(B)** Our fine-grained weed recognition method with contrastive loss. Bluer colors indicate larger numerical values. C1–C8 denote the abbreviations of maize seedling and weeds.

As shown in **Table 3**, we observe that with the help of contrastive loss, the method obtains a big performance gain. Specifically, it increases the accuracy from 97.96% to 99.18% for our method. A similar result can be seen by introducing contrastive loss in which the accuracy is boosted from 97.35% to 97.75% for EfficientNetV2. The experimental results illustrate that contrastive loss is helpful to improve recognition performance. This is because contrastive loss can effectively improve the distinguishing ability by enlarging the distance of feature representations between similar maize seedlings and weeds. In addition, we show the confusion matrices of our method on the testing set in **Figure 8**. It can be seen that major misclassifications occur within these categories with similar visual appearance and texture, such as Maize seedling and Sedge. However, with contrastive loss, the number of misclassified samples in the recognition of Maize seedling and Sedge dropped from 4 and 3 to 1, respectively. This phenomenon further proves the advantage of contrastive loss in distinguishing subtle morphological differences between maize seedlings and weeds.

**Table 3:** Ablation study on contrastive loss on the MWFI (maize/weed field image) dataset.

Methods	Contrastive loss	Accuracy/%
EfficientNetV2	Without	97.35
EfficientNetV2	With	97.75
Our method	Without	97.96
Our method	With	99.18

### 5.4 Visualization analysis

**Figure 9:**
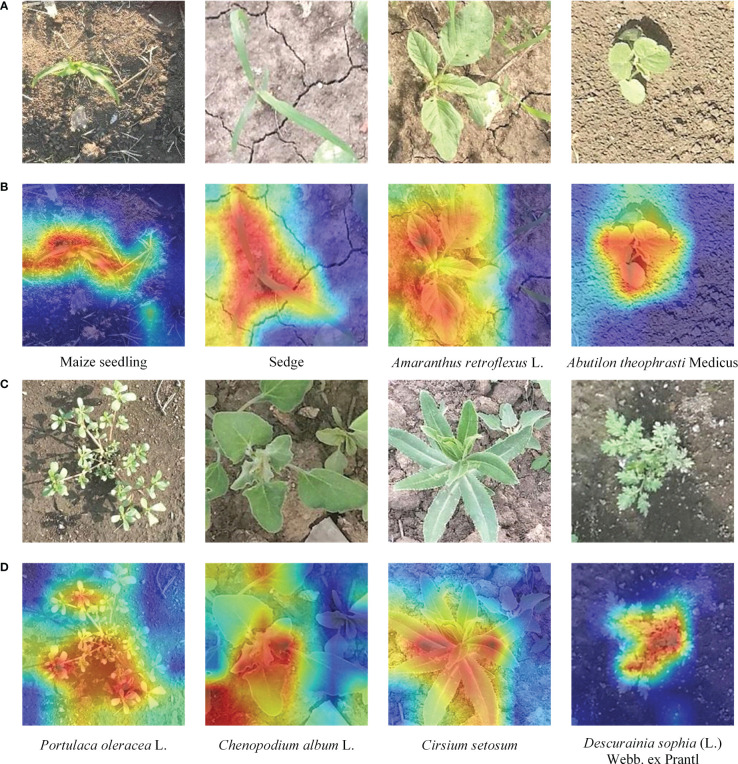
Visualization results of our fine-grained weed recognition method. **(A)** The original images of the first four categories. **(B)** The visualization outputs of the first four images. **(C)** The original images of the last four categories. **(D)** The visualization outputs of the last four images. The darker the color, the more critical the region is to the recognition result.

The key to the fine-grained image classification task is to pay attention to the most discriminative part of the image. To better understand the parts of the plant emphasized by our proposed method, we used the gradient-weighted class activation heatmap to visualize our method (Selvaraju et al., 2017). The correct labels and output feature maps of the feature extraction network were used in generating the activation distribution. We randomly sample one image from each category of the testing dataset and visualize them as shown in **Figure 9**.

From **Figure 9**, we can find that our method can focus well on the most important parts of the maize seedling and weed images and meanwhile ignore some background regions. For example, in the first and second column of **Figure 9B**, there are high activation values in the region of the leaf in the visualization results of Maize seedling and Sedge images, which can significantly help to distinguish weeds from crops with similar morphology. For example, in the second and third column of **Figure 9D**, it can be seen that the overall heatmap obtained by our method can accurately cover the area where the target category is located even if there is interference from other categories of weeds in the images.

### 5.5 Experiment on the DeepWeeds dataset

Besides the MWFI dataset, we also conduct experiment on the DeepWeeds dataset to further evaluate the effectiveness of our method. The DeepWeeds dataset includes 17,509 images of eight weed categories and a negative category (other plants except for 8 weeds) taken in rangeland environments, and **Supplementary Figure 2** provides an example image for each category. This dataset is split following the setup in Olsen et al. (2019); 60% of the dataset is used for training, 20% is used for validation, and the remaining 20% is used for testing. The performance of our method and existing methods are comparatively reported in **Table 4**. It can be observed that our proposed method achieves the best performance in terms of accuracy, precision, and recall. It is also noted that GWN-DenseNet202 achieves higher precision and recall than other compared methods. This is because GWN-DenseNet202 can locate the key parts of weed images to distinguish weeds with high appearance similarity. Although GWN-DenseNet202 has the same precision as our method, its recall is 0.3% lower than our method. This phenomenon shows that capturing the discriminative parts of images is useful to improve weed recognition performance, and our method is more effective than GWN-DenseNet202 for capturing key parts.

**Table 4:** Performance comparison of different methods on the DeepWeeds dataset.

Methods	Dataset split (Train : Valid:Test)	Evaluation metric	Best value/%
Inception-v3 (Olsen et al., 2019)	6:2:2	Recall	95.1
		Precision	95.1
ResNet-50 (Olsen et al., 2019)	6:2:2	Recall	95.7
		Precision	95.7
GWN-DenseNet-202 (Hu et al., 2020)	6:2:2	Recall	98.1
		Precision	99.18
ResNet-50-kd (Peng and Wang, 2021)	6:2:2	Accuracy	96.9
SWAV-xResNet-34 (Güldenring and Nalpantidis, 2021)	6:2:2	Recall	96.6
DenseNet-201-KNN (Huertas-Tato et al., 2022)	7:1:2	Precision	94.7
Our method	6:2:2	Accuracy	98.6
		Recall	98.4
		Precision	98.2

**Figure 10:**
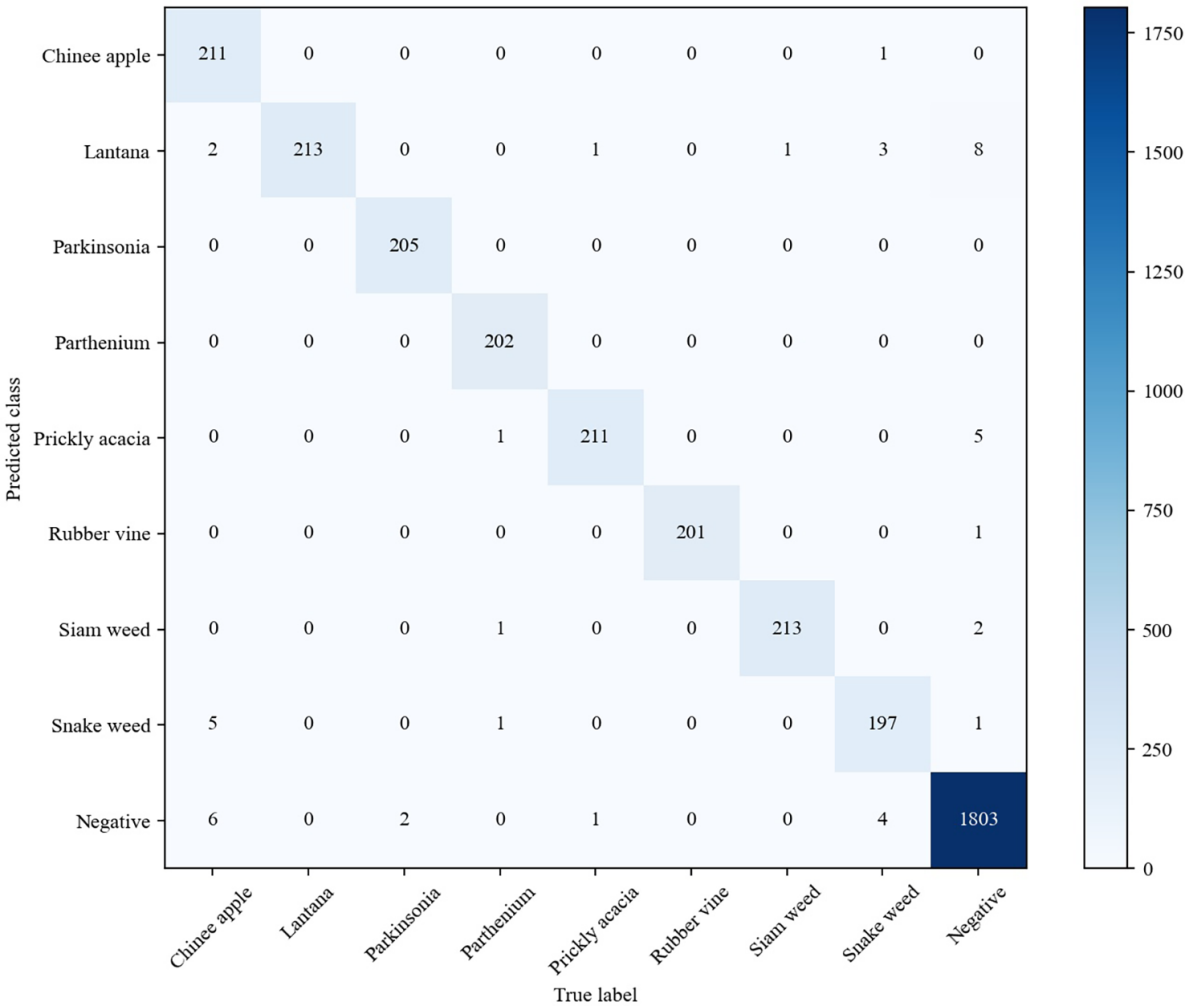
Confusion matrix of our fine-grained weed recognition method on the DeepWeeds dataset.

**Figure 10** shows the confusion matrix of our method on DeepWeeds. It can be seen that our method provides outstanding performance for each category, even though some categories of weeds are morphologically similar. Although Chinee apple and Snake weed are easily confused in the DeepWeeds dataset, our method is able to reduce misclassifications between them effectively. Specifically, one Chinee apple sample was misclassified as Snake weed and five Snake weed samples were misclassified as Chinee apple by our method. Compared with the GWN-DenseNet202, our method is superior in distinguishing Chinee apple and inferior in distinguishing Snake weed. The analysis of the confusion matrices further demonstrates that our method is a powerful architecture for weed recognition.

## 6 Conclusions

This study presents a fine-grained recognition method for classifying maize seedlings and weeds in the field. We exploit the Swin Transformer network to capture the most discriminative regions in maize seedling and weed images. Meanwhile, our method further expands the feature differences of similar image samples between different categories by applying contrastive loss. In addition, we use the two-stage transfer learning strategy to reduce requirements for the amount of annotated data and improve recognition accuracy. The experimental results on the MWFI and DeepWeeds datasets show the effectiveness of the proposed method. Moreover, the average recognition time of our method is only 56 ms with CPU, which can meet the real-time requirement of field weed control. In this study, we presented the idea of learning local and global information in weed and crop images by using the Swin Transformer network, which is beneficial for distinguishing the images of appearance similarity and can be applied to other agriculture image recognition. In our future work, we plan to evaluate the effectiveness of the transformer-based methods in multi-label prediction, object detection, and semantic segmentation of weeds. Moreover, we will improve the real-time ability of our proposed method and study the deployment in edge devices.

## Data availability statement

The raw data supporting the conclusions of this article will be made available by the authors, without undue reservation.

## Author contributions

YW, SZ, and BD: designed the study, conducted the survey, and trained the model. SY and HS: collected and processed the data. YW, SZ, and BD: writing the original draft preparation. All authors contributed to the article and approved the submitted version.
