# Supplementary material for: Fine-grained weed recognition using Swin Transformer and two-stage transfer learning
Source: Front Plant Sci. 2023 Mar 13;14:1134932. doi: 10.3389/fpls.2023.1134932 (PMC10040655; doi:10.3389/fpls.2023.1134932)
Supplement: Supplementary file 1 [file DataSheet_1.docx]

Supplementary Material

Fine-grained weed recognition using Swin Transformer and two-stage transfer learning

Yecheng Wang, Shuangqing Zhang, Baisheng Dai*, Sensen Yang, Haochen Song

**Correspondence:** Baisheng Dai [bsdai@neau.edu.cn](mailto:bsdai@neau.edu.cn)

| Supplementary Table 1. The distribution of the training set and testing set images. | | | | |
| --- | --- | --- | --- | --- |
| Full name | Class | Original training set | Final training set | Testing set |
| Maize seedling | C1 | 220 | 600 | 94 |
| *Cyperus rotundus* L. | C2 | 157 | 600 | 68 |
| *Amaranthus retroflexus* L. | C3 | 230 | 600 | 98 |
| *Abutilon theophrasti* Medicus | C4 | 85 | 600 | 37 |
| *Portulaca oleracea* L. | C5 | 70 | 600 | 30 |
| *Chenopodium album* L. | C6 | 132 | 600 | 56 |
| *Cirsium setosum* | C7 | 128 | 600 | 55 |
| *Descurainia sophia* (L.)  Webb. ex Prantl | C8 | 120 | 600 | 52 |

| Supplementary Table 2. The experimental results of different methods trained on whole Plant Seedlings dataset. | | | | |
| --- | --- | --- | --- | --- |
| Methods | Accuracy/% | Precision/% | Recall/% | F1 Score/% |
| VGG-16 | 99.04 | 98.85 | 99.00 | 98.92 |
| ResNet-50 | 99.19 | 98.99 | 99.23 | 99.11 |
| DenseNet-121 | 99.28 | 99.12 | 99.28 | 99.20 |
| SE-ResNet-50 | 99.10 | 99.03 | 99.13 | 99.08 |
| EfficientNetV2 | 99.24 | 99.14 | 99.28 | 99.21 |
| Swin Transformer | 99.60 | 99.61 | 99.59 | 99.60 |


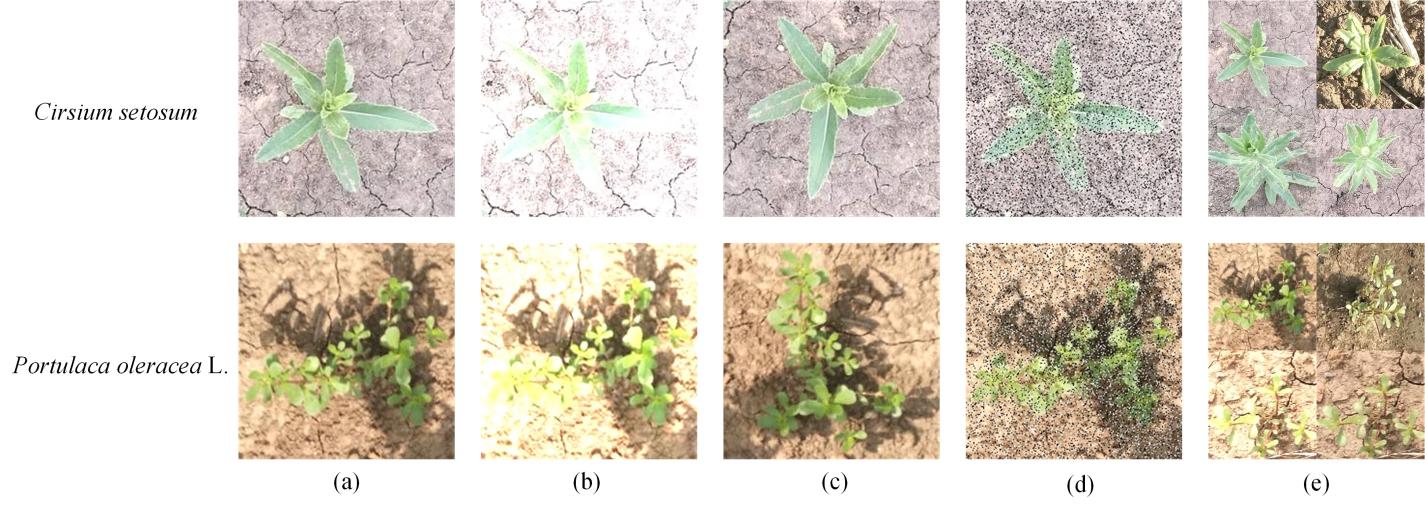


Supplementary Figure 1. Example results of different data augmentation techniques. (a) Original image, (b) Brightness enhancement (range from1.1 to 1.5), (c) Random rotation (from 0°to 360°with a step of 90°), (d) Salt and pepper noise, (e) Mosaic.


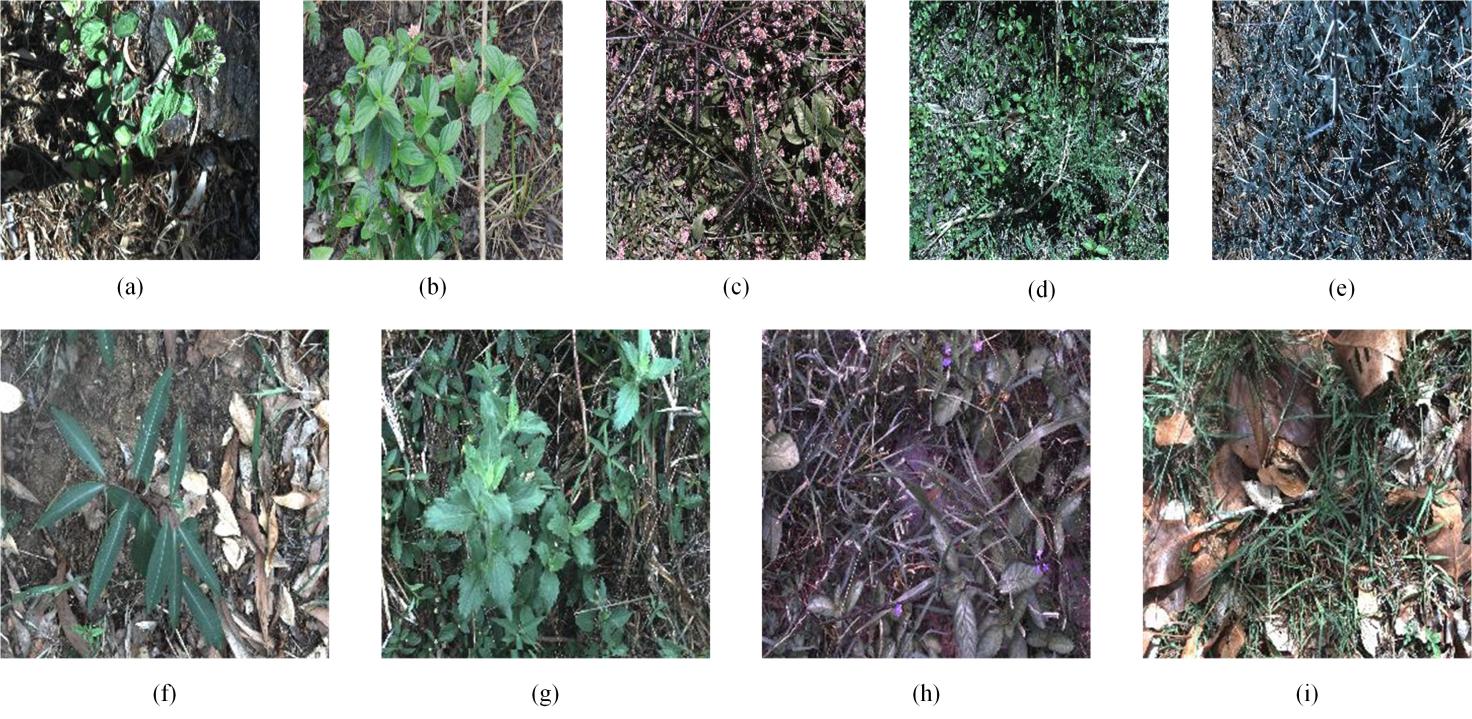


Supplementary Figure 2. Example images of the 9 categories in the DeepWeeds dataset. (a) Chinee apple, (b) Lantana, (c) Parkinsonia, (d) Parthenium, (e) Prickly acacia, (f) Rubber vine, (g) Siam weed, (h) Snake weed. (i) Negative.
